# Does the COVID-19 personal protective equipment impair the surgeon’s performance?

**DOI:** 10.1007/s00402-022-04416-2

**Published:** 2022-03-19

**Authors:** Jan P. Kolb, Annika Hättich, André Strahl, Tim Rolvien, Jan K. Hennigs, Alexej Barg, Karl-Heinz Frosch, Maximilian J. Hartel, Carsten Schlickewei

**Affiliations:** 1 Department of Trauma and Orthopaedic Surgery, University Medical Center Hamburg-Eppendorf, Martinistrasse 52, 20246 Hamburg, Germany; 2 Department of Pneumology, University Medical Center Hamburg-Eppendorf, Hamburg, Germany; 3 Department of Trauma Surgery, Orthopaedics and Sports Traumatology, BG Hospital Hamburg, Hamburg, Germany; 4 Department of Orthopaedics, University of Utah, Salt Lake City, USA

**Keywords:** COVID-19, SARS-CoV-2, Personal protective equipment, Surgical procedure, Surgeon’s mental and physical performance, Fatigue

## Abstract

**Introduction:**

Despite increasing vaccination rates, new viral variants of SARS-CoV-2 (severe acute respiratory syndrome coronavirus type 2) are advancing the COVID 19 (coronavirus disease 2019) pandemic and continue to challenge the entire world. Surgical care of SARS-CoV-2 positive patients requires special protective measures. We hypothesized that "COVID-19" personal protective equipment (PPE) during surgery of SARS-CoV-2 positive or potentially positive patients would negatively affect the surgeon and thus the surgical outcome.

**Materials and methods:**

Ten experienced trauma surgeons participated in the study. Each surgeon performed two simulated surgeries of a distal tibial fracture on a Sawbone® under standardized conditions either wearing regular PPE or special COVID-19 PPE. Baseline values at rest were acquired for heart rate, blood pressure, saturation of peripheral oxygen (SpO_2_), respiratory rate and capillary blood gas (CBG) analysis including capillary partial pressure of oxygen (pO_2_) and carbon dioxide (pCO_2_), followed by four different standardized tests of attentional performance (TAP). Subsequently, the surgeon performed the first surgery according to a randomly determined order, with regular or COVID-19 PPE conditions in an operation theatre. After each surgery vital signs were acquired and CBG and TAP were performed again.

**Results:**

In our simulated surgical procedure heart rate, respiratory rate, systolic and diastolic blood pressure did not show relevant differences. Percutaneously measured SpO_2_ decreased with additional layers of PPE, while CBG parameters were not affected. TAP tests showed a significant impairment of attention if PPEs were compared to the baseline, but both PPEs had similar results and no meaningful differences could be measured.

**Conclusions:**

According to our results, for surgical procedures additional PPE required during COVID-19 pandemic does not relevant affect the surgeon’s mental and physical performance. Surgeries under COVID-19 PPE conditions appear safe and do not increase patient risk.

**Level of evidence:**

Level I.

## Introduction

The COVID-19 pandemic continues to challenge the entire world [2, 3, 5, 6, 11, 14, 19]. While global vaccination strategies are currently implemented, new viral variants of SARS-CoV-2 continue to emerge and contagion rates remain high across the globe [17, 23]. High infection rates inevitably constitute an increased likelihood of SARS-CoV-2-positive patients requiring emergency surgery or non-deferrable interventions. In addition, there are countless procedures, where surgery must be performed due to medical emergency, even though there was insufficient time to safely determine the patient's COVID-19 status and rule out a risk of infection for the medical staff. As such, additional precautions must be taken including the use of special personal protection equipment (PPE) to prevent airborne transmission of the SARS-CoV-2 not only for personal protection of healthcare workers but also to maintain a highly functioning health care system in general [1, 2, 10, 16, 20].

The WHO (World Health Organization) ad hoc COVID Infection Prevention and Control Guidance Development Group (COVID IPC GDG) recommends protective masks with additional protection (e.g., N95, N99, FFP2, FFP3, or equivalent). High infection rates among healthcare workers (vaccinated, unvaccinated, or recovered of COVID-19) can only be prevented through the use of appropriate PPE. Thus, in addition to standard PPE at least three layers of partially fluid-repellent and waterproof clothing including a liquid-repellent surgical mask on top of an FFP2 mask and medical goggles are required for standard operative procedures on SARS-CoV-2 positive patients by local authorities. Trauma and orthopaedic surgeons wear a lead gown as extra protective equipment.

We hypothesized that the additional PPE measures, meant to protect the staff against COVID-19 in an operation theatre, leads to substantially increased physical and mental stress resulting in decreased alertness in surgeons. This might have direct influence on the surgeon's attention, accuracy of surgical steps performance, the intraoperative patient safety, and negatively affect the surgical outcome. In this study, we explored these questions simulating the surgical treatment of a distal tibial plafond fracture using a saw-bone model in a regular operation theatre either in “standard” or “COVID-19” PPE. To measure the surgeon's workload, vital signs, and capillary blood gas (CBG) analysis were performed before and after simulated surgery. The attentional function, as a basic prerequisite for general performance and alertness, was assessed with four different standardized attention subtests from the test of attentional performance (TAP) [26].

## Materials and methods

### Study design

This study was conducted in accordance with the Declaration of Helsinki and the Guidelines for Good Clinical Practice [12]. The Medical Board of Hamburg (Ärztekammer Hamburg 15022021DH) approved this study. Ten in traumatology fellowship trained trauma surgeons (9 males, 1 female) with a mean age of 37.2 ± 3.9 years participated in the study (Table 1). All surgeons voluntarily agreed to participate in this study were informed comprehensively about the study and gave written consent. The data collected were stored and analyzed anonymously. Mean years of experience were 11 years (range, 4–15). Each surgeon performed two simulated surgeries of a distal tibial plafond fracture using Sawbone® under realistic and standardized conditions either wearing regular (“standard”) PPE or special COVID-19 PPE. Surgery simulation took place in a regular operation theatre with a lamina air flow system under constant room temperature (18 °C). The study was carried out during normal working hours.

**Table 1:** Demographics and vital baseline parameters of study participants

Parameter	No. of study participant										Mean ± SD or n (%)
	1	2	3	4	5	6	7	8	9	10	
Demographic characteristics											
Gender	m	m	m	m	m	f	m	m	m	m	9/10 (90.0%)
Age (years)	37	42	38	39	39	33	29	41	38	34	37.0 ± 3.9
Height (cm)	195	189	185	179	182	173	190	193	180	185	185.1 ± 6.8
Weight (kg)	100	100	86	73	93	65	80	86	72	84	83.9 ± 11.8
BMI (kg/m^2^)	26.3	28.0	25.1	22.8	28.2	21.7	22.2	23.1	22.2	24.5	24.4 ± 2.4
Smoker	no	yes	yes	yes	no	yes	no	no	no	no	4/10 (40.0%)
Pack years	10	10	15	6	12	5	90	0	0	0	5.8 ± 5.7
Vital parameters											
Heart rate (bpm)	73	82	71	100	61	58	63	82	59	57	70.6 ± 14.0
Respiratory rate	12	16	16	15	12	10	18	12	12	16	13.9 ± 2.6
Systolic blood pressure (mmHg)	124	141	157	138	144	116	128	156	128	145	137.7 ± 13.6
Diastolic blood pressure (mmHg)	77	92	104	90	100	81	69	89	91	94	88.7 ± 10.5
Oxygen Saturation (SpO_2_ in %)	100	95	99	99	99	99	99	99	96	99	98.4 ± 1.6

*BMI* body mass index, *bpm* beats per minute, *f* female, *m* male, *n* number of cases, *SD* standard deviation, *SpO*_*2*_ Oxygen saturation

The order of the surgeons and the test setting was determined at random by drawing two sealed envelopes. The first envelope determined the order in which the surgeons performed the surgery (numbers 1 to 10). To achieve a statistically balanced distribution, the second envelope determined whether the surgeons performed the first operation with standard PPE in an operation theatre or with COVID-19 PPE. For standard and the COVID-19 PPE, current standard guidelines at our University Medical Center Hamburg–Eppendorf were followed as described below (Table 2).

**Table 2:** “Standard “and “COVID-19” personal protective equipment (PPE) in an operation theatre

“Standard” PPE	“COVID-19” PPE
Surgical waterproof boots	Surgical waterproof boots
Surgical scrub suit	Surgical scrub suit
Surgical hood with ties	Surgical hood with ties
Waterproof X-ray protection apron	Waterproof X-ray protection apron
Surgical face mask	FFP2 mask + surgical face mask
–	Medical safety googles
–	Unsterile waterproof protective gown
Waterproof sterile gown	Waterproof sterile gown
Double sterile gloves	Double sterile gloves

*FFP* filtering facepiece, *PPE* personal protective equipment

Before surgery, the following parameters were collected from each surgeon: age, sex, body weight, height, smoking status and history, and previous pulmonary diseases. Baseline values at rest were acquired for heart rate, blood pressure (Riva Rocci method), peripheral oxygen saturation (SpO_2_), respiratory rate and capillary blood gas (CBG) analysis including capillary partial pressure for oxygen (pO_2_) and carbon dioxide (pCO_2_), followed by four different standardized subtests of TAP (see below). Subsequently, the surgeon performed the first surgery according to the randomly determined order, with standard or COVID-19 PPE conditions in an operation theatre.

As basic PPE each surgeon wore surgical waterproof boots, a surgical scrub suit, a surgical hood with ties, a waterproof X-ray protection apron and one surgical face mask. Basic PPE in the operation theatre consisted of additional sterile gown and double sterile gloves, after surgical hand preparation. For COVID-19 PPE the surgical scrubbing routine was adapted to the most recent protocol being used.

In addition to basic PPE the surgeon wore a FFP2 mask under the surgical face mask (double masking), medical safety googles, and a non-sterile fluid-repellent long gown over the waterproof X-ray protective apron. After surgical hand preparation the surgeon was provided with a sterile gown and double sterile gloves.

**Fig. 1:**
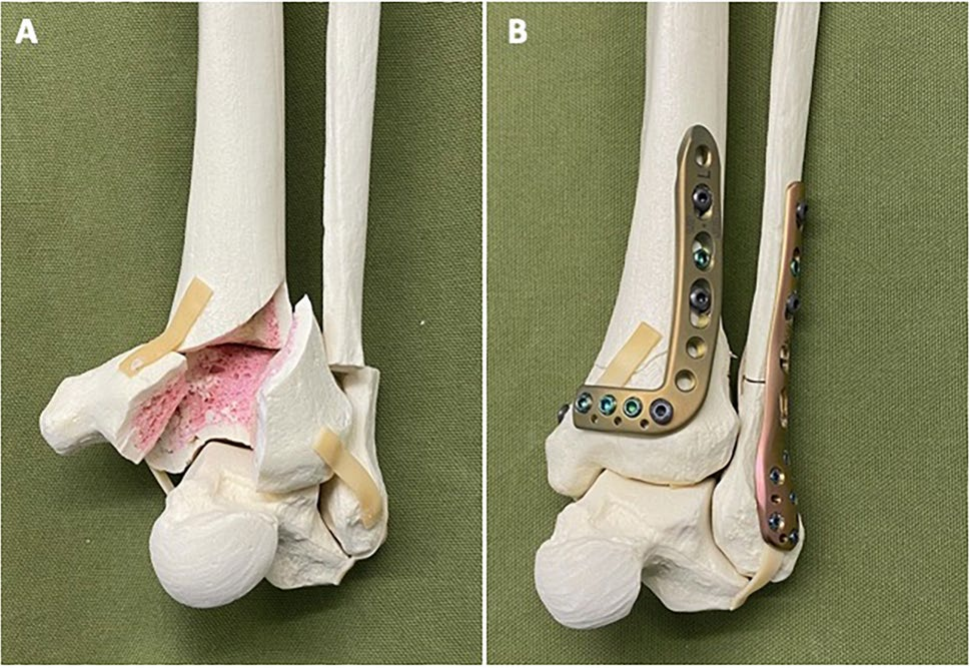
Distal tibial plafond fracture. **A** Preoperative and **B** after reduction and stabilization (Locking Compression Plate (LCP® DePuy Synthes)

The distal tibial plafond fracture was reduced and stabilized with standard instruments and an angular stable plate osteosynthesis (Locking Compression Plate (LCP® DePuy Synthes), Fig. 1A, B). A standard operating time of 30 min was set for each procedure. After the time, the surgery was terminated regardless of the surgical progress. Vital signs, CBG and TAP were determined again. The whole procedure was repeated for the second surgery using the other PPE scheme. Following every simulated surgery, surgeons reported subjective stress and exhaustion under standard and COVID-19 conditions on a 11-item numerical scale from 0 to 10. (0 = not exhausting at all to 10 = maximum exhausting) [7].

### Vital signs measurement

Vital signs measurement was carried out using a monitor system (Draeger Infinity® Delta monitor) for non-invasive blood pressure measurements (Riva Rocci method) on the upper arm (BP), heart rate (beats per minute, bpm) and percutaneous oxygen saturation (SpO_2_ in percent, via pulse oximeter). The respiratory rate (RR) was counted over a period of 60 s.

### Capillary blood gas (CBG) analysis measurement

CBG was performed as arterialized capillary blood gas analysis on the earlobe. To reduce venous mixing of the blood sample, the earlobe was rubbed with a vasodilating ointment (Finalgon®, Boehringer Ingelheim) before puncture and blood draw into heparinized glass capillaries. CBG analysis was also performed (Radiometer ABL90, Flex). Data for oxygen partial pressure (pO_2_) and carbon dioxide partial pressure (pCO_2_), pH value and hydrogen carbonate (HCO_3_ ) were extracted.

### Test of attentional performance

Participant’s attention was assessed with four subtests of the standardized computer-assisted TAP Version 2.3.1: Alertness,  Go/No-Go, Flexibility and Divided Attention [27, 28]. The alertness subtest measures the reaction time in milliseconds (ms) to a given visual stimulus of the screen with and without an auditory warning signal. When the stimuli are displayed, a button must be pressed as quickly as possible. The Go/No-Go subtest assessed the participant’s inhibitory control. The test presents sequential ether a target or a non-target stimulus, whereby the button only must be pressed on target stimuli. Both, reaction time for correct responses and the number of errors (wrong marks) are recorded. The Flexibility subtest is a "set shifting" task. To the right and left side of the screen an angular and a round figure are presented simultaneously. During the task, the target stimuli change constantly from angular to round figure and participants must chose with a left and right button the complementary target stimulus (e.g., in the sequence: “round”—“angular”—“round”—“angular” etc.). Divided Attention presents a visual and an auditory dual task simultaneously. The visual task involves pressing a button when four crosses form a square on a matrix, while in the auditory test a button must be pressed when the same tone is played twice in a row. Reaction time, the number of errors (wrong marks), and the number of missing are recorded.

All participants were tested individually in the surgery room at three timepoints using a computer in a random test sequence. The first test was performed before the start of the simulation surgery (baseline). Furthermore, two tests were carried out after each surgery. The test instructions were presented in writing and explained in addition, if necessary, by an experienced supervising psychologist. Before each subtest was performed, a trial run was completed to identify comprehension and execution problems.

### Statistical analysis

The required sample size for this study was calculated based on TAP manual. The expected effect size was estimated from the minimal clinical important difference (MCID) and the standard deviation (SD) of the norm sample for the Divided Attention subtest. The Divided Attention subtest was chosen for this purpose as it reflects the ability needed to divide attention to simultaneously ongoing processes as required during complex surgery. MCID, as patient derived scores reflecting meaningful changes, was 222.2 ms for reaction time of visual divided attention and 180.5 ms for auditory divided attention. Considering the SD of the norm sample of the Divided Attention subtest, a large effect size of 1.44, respectively, and 1.48 could be derived from these values. With an alpha risk of 0.05 and a statistical power of 0.8, a total sample size of n = 5 samples was calculated. To account for the large effect size calculated, it was decided to double the sample size to n = 10, during the design of the study.

All data were analyzed using IBM SPSS Statistics version 26.0 (IBM, Armonk, NY) and GraphPad Prism 9 (GraphPad Software, La Jolla, CA). Continuous variables are expressed as mean ± SD, while categorial variables are expressed as number and percentage. Shapiro–Wilk normality test and Kolmogorov–Smirnov test were performed to determine if the data were normally distributed. Spearman coefficient was used for correlation of nonparametric data. The median reaction times and errors were evaluated using one-way repeated measures ANOVAs for normally distributed data or Friedman-test for non-normally distributed data to determine whether significant differences exist among measuring conditions. The level of significance for all tests was set at *P* ≤ 0.05.

## Results

### Vital signs and capillary blood gas analysis

Vital signs were collected at baseline, before and after the simulated surgeries. We did not find any significant differences in systolic and diastolic blood pressure, heart rate or respiratory rate between individuals at baseline or across PPE conditions. SpO_2_ under room air showed a slight decrease with additional PPE measures but no statistically significant differences between any two of the three conditions were detected. In addition, while we detected minor non-significant changes in the CBG parameters pO_2_, pCO_2_ and pH across PPE conditions, all remained well within normal limits (Table 1).

### Subjective experience of stress

Performing surgery with COVID-19 PPE was considered statistically more distressing (4.5 ± 1.3) than with standard PPE (2.3 ± 0.7; *P* = 0.005). Moreover, a non-significant trend towards lower pO_2_ values (*r* = – 0.354, *P* = 0.316) and higher pCO_2_ values (*r* = 0.617, *P* = 0.058) in the BGA with higher subjective stress during surgery while wearing the "COVID-19″ PPE was observed.

### Test of attentional performance

**Fig. 2:**
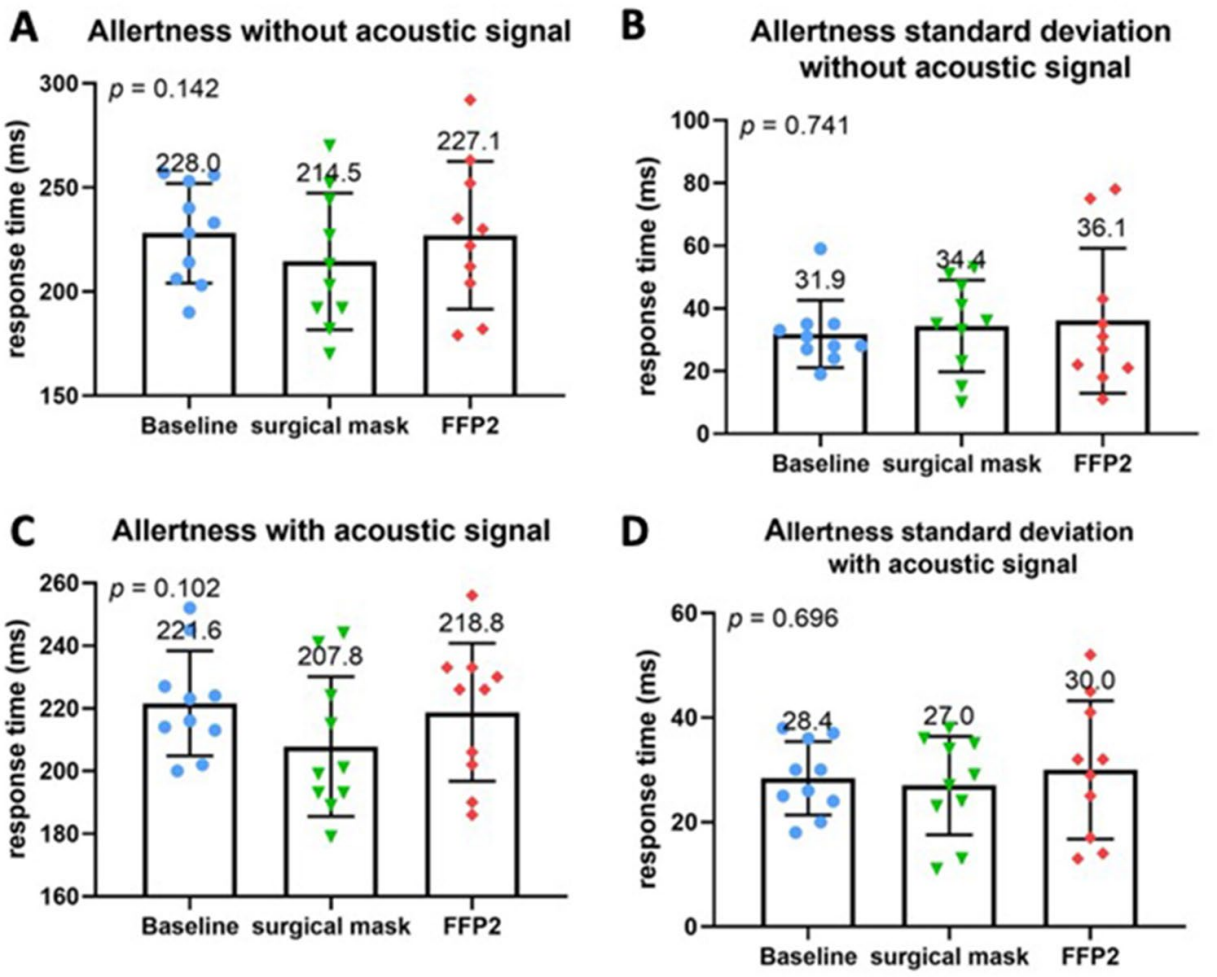
Test of attentional performance—subtest alertness. Comparison of response times in milliseconds between the three investigated groups at baseline, operating with surgical mask, and operating with FFP2 mask in the test results **A** Alertness without an acoustic signal, **B** standard deviation of Alertness without an acoustic signal, **C** Alertness with an acoustic signal, and **D** standard deviation of Alertness with an acoustic signal

**Fig. 3:**
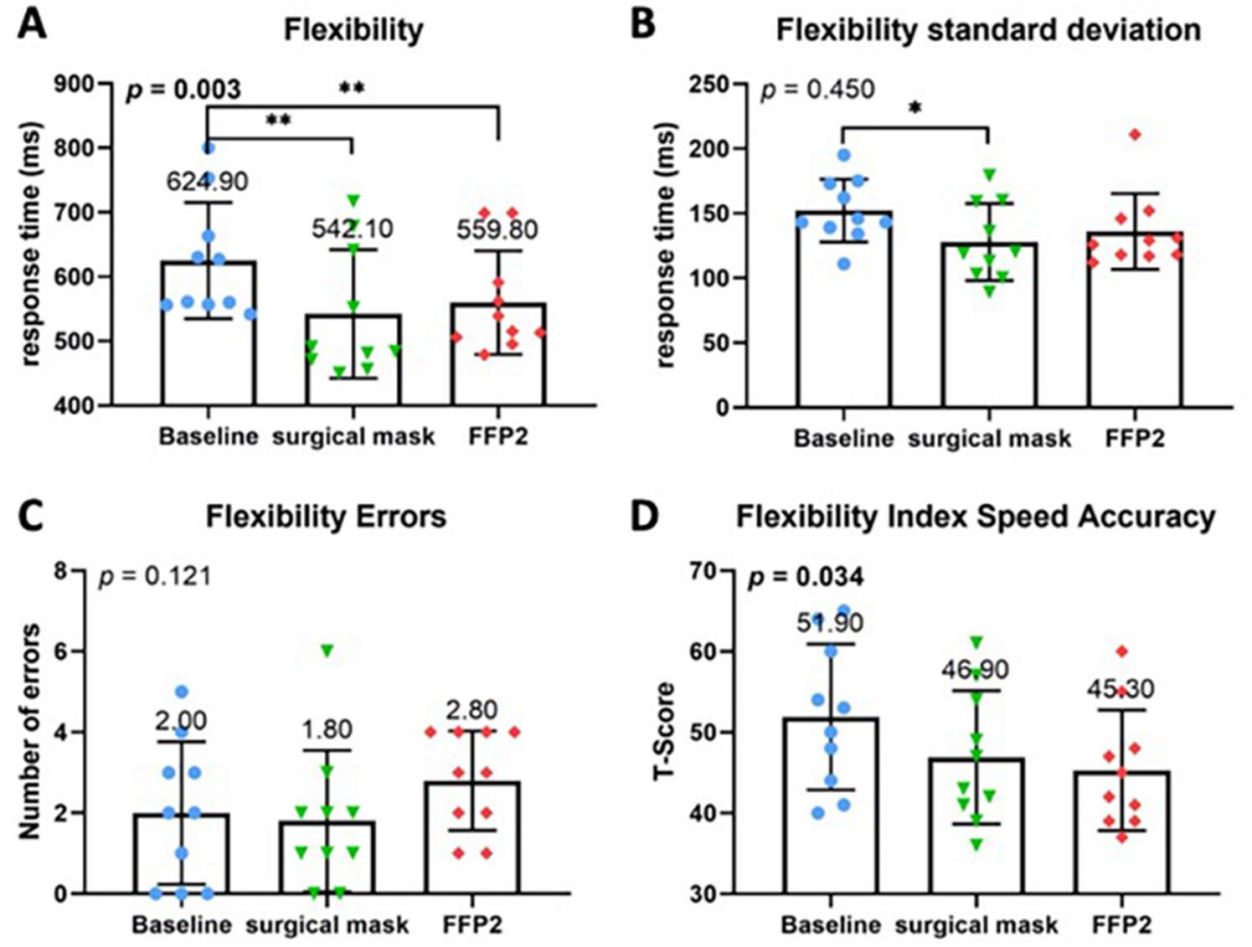
Test of attentional performance—subtest flexibility. Comparison of response times in milliseconds between the three investigated groups at baseline, operating with surgical mask, and operating with FFP2 mask in the test results. **A** Flexibility reaction time, **B** standard deviation of Flexibility reaction time. **C** Comparison of number of errors made during performance of the test. **D** Comparison of the standardized T-Score between the three investigated groups according to the flexibility speed–accuracy index

**Fig. 4:**
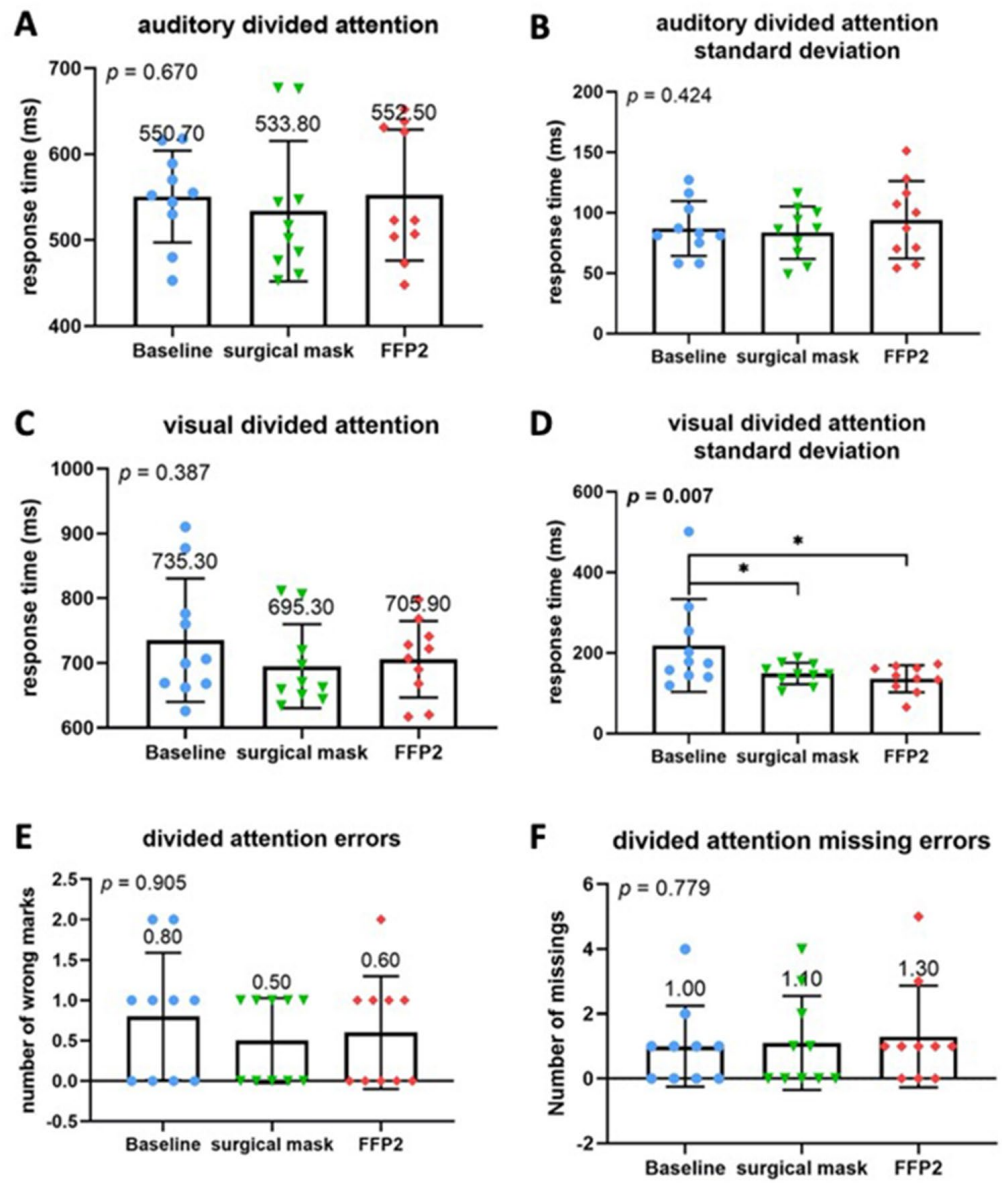
Test of attentional performance—subtest divided attention. Comparison of response times in milliseconds between the three investigated groups at baseline, operating with surgical mask, and operating with FFP2 mask in the test results. **A** Auditory divided attention reaction time, **B** standard deviation of auditory divided attention reaction time, **C** visual divided attention reaction time, **D** standard deviation of visual divided attention reaction time. **E** Comparison of number of errors made during performance of the divided attention test. **F** Comparison of number of missing errors made during performance of the divided attention test

**Fig. 5:**
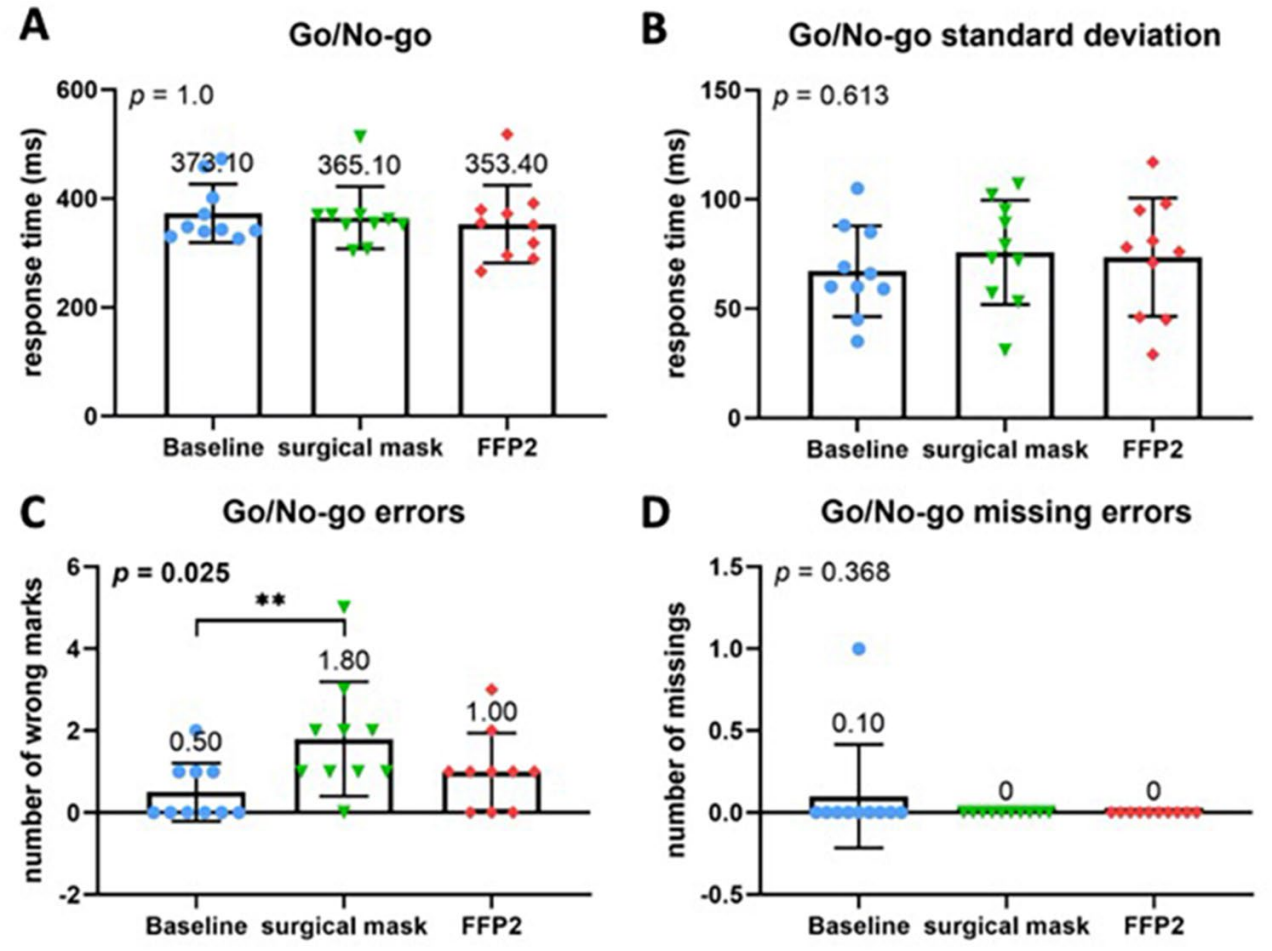
Test of attentional performance—subtest Go/No-Go-Test. Comparison of response times in milliseconds between the three investigated groups at baseline, operating with surgical mask, and operating with FFP2 mask in the test results. **A** Go/No-Go reaction time, **B** standard deviation of the Go/No-Go reaction time. **C** Comparison of number of errors made during performance divided attention test. **D** Comparison of number of missing errors made during performance of the Go/No-Go test

The alertness subtest did not reveal any significant differences regarding the mean values or standard deviations between baseline and either standard or COVID-19 PPE (Fig. 2A–D). However, other TAP subtests showed a significant difference for both PPEs compared to the baseline. Namely, both standard and COVID-19 PPE had a significant impact on reaction time in the flexibility subtest (*p* = 0.003) with a strong negative effect on reaction time (Fig. 3A, B). Although no significant differences were found regarding the absolute number of error rates (Fig. 3C), compared to baseline speed–accuracy-index was significantly decreased across all PPE conditions (*P* = 0.034) (Fig. 3D). No difference between standard and COVID-19 PPE was detected. In the divided attention subtest, there were no differences for auditory or visual divided attention, or divided attention errors between baseline, standard, and COVID-19 PPE (Fig. 4A–F). Despite no change in reaction times in the Go-No–Go Go/No-Co subtest (Fig. 5A, B), the error rate increased when PPE was used (*P* = 0.025) (Fig. 5C), regardless of PPE type (*P* = 0.081). In summary, results in all four TAP subtests were partially reduced but within the standard range, either for standard or COVID-19 PPE (Figs. 2, 3, 4, 5).

## Discussion

In the present study, we investigated whether the additional PPE measures as extra infection prevention during the COVID-19 pandemic correlated with increased physical and psychological distress in trauma surgeons. During the simulated surgical procedure heart rate, respiratory rate, systolic and diastolic blood pressure did not show any relevant differences. Percutaneously measured SpO_2_ decreased with additional layers of PPE, while CBG parameters were not affected. Importantly, all measured values (vital signs and CBG) remained constantly within their respective reference range and are, therefore, likely without relevant physiological impact [13, 21, 22]. In contrast, a recent study in oral surgeons reports much more pronounced PPE-related differences in heart rate and oxygenation even when performing short surgical procedures [18]. In keeping with possible underlying confounders, in the present study surgeons were older and likely more experienced. This in line with reports showing heightened intraoperative stress levels in less experienced surgeons [9, 24].

In addition to physiological impact, PPE-related mental stress was assessed using TAP tests and a self-reported 10-item stress indicator scale. TAP tests showed a significant impairment of attention. Both standard and COVID-19 PPE had a significant impact on the reaction time in the flexibility subtest and a strong negative effect on the reaction time. Although no significant differences were found regarding the errors in this subtest, there was a significant decrease in the speed–accuracy-index across all conditions. For the Go/No-Go subtest, here, no change in the reaction times were documented, but significantly more errors were recorded for both PPE groups.

The results of the TAP test show that even wearing "normal" PPE including X-ray protection leads to a significant impairment of attention. This is also evident for COVID-19 PPE. Both PPE in combination with X-ray protection lead to a negative influence on reaction time, while the error rate remains the same. This is in contrast to the results of the  Go/No-Go test, which could not prove any impairment of reaction time, but showed a significantly higher error rate for PPE groups. We have no explanation for the contradictory effect on reaction time in the two tests. However, the significant increase in error rate in the Go/No-Go test could be explained by the complexity of the test after the simulated surgery. Nevertheless, the studies show that both PPE have a significant effect on the surgeon. However, this effect has no significant impact in the direct comparison of the two PPE groups against each other.

Due to the SARS-CoV2 pandemic CDC and ECDC widely recommend PPE, not only in hospitals. Despite rising vaccination rates viral variants of SARS-CoV-2 differ from conventional virus variants in their pathogen characteristics, such as transmissibility, virulence, or susceptibility. Some variants remain infectious even to vaccinated or recovered individuals. Some variants remain infectious even for vaccinated or recovered individuals. Appropriate "COVID-19" PPE and adequate rest periods continue to be considered key factors in protecting patients and medical staff and to reduce the risk of negative psychological consequences [8]. Fikenzer et al. reported reduction of ventilation, cardiopulmonary exercise capacity and comfort by surgical masks and highly impairment by FFP2/N95 face masks [4].

We hypothesized that these could lead to increased physical stress, reduced air supply, and decreased alertness in surgeons, which may have a direct influence on the surgeon's attention, the patient safety and negatively affect the surgical outcome. In comparison the oxygen content measured percutaneously under "COVID-19″ PPE was lower when compared to the baseline and "standard" PPE conditions but without falling below the normal value or statistical relevance. Subjectively, surgery was perceived as more stressful under "COVID-19″ conditions than under "standard" conditions. TAP tests showed a significant impairment of attention if the PPEs were compared to the baseline, but both PPEs had similar results and no differences for both PPEs could be measured. Although these results imply an impact of PPE, we did not find any relevant differences between the two PPEs. Although the measured values showed a negative trend for "COVID-19″ PPE, normal values could always be measured here as well. This suggests that surgical management of a SARS-CoV-2 positive patient in emergency situations may lead to equally good results as in non-infected patients from surgery performance point of view. In our study, we did not find any relevant differences between "standard" PPE and "COVID-19″ PPE.

The presented study has some limitations. The simulated surgeries are a major limitation of our study, even though the entire setting from operating room to the instruments used correspond to real surgeries. However, to create standardized conditions, to exclude the influence of different types and lengths of surgery, and to avoid putting real patients at unnecessary risk, we chose the setting described. Procedure time was rather short with a total of 30 min. However, emergency procedures are primarily to stabilize the patient (e.g., use of external fixators for the stabilization of polytrauma patients) and should, therefore, generally be kept as short as possible before the patient can be transferred to an intensive care unit. In addition, blood was taken from the earlobe for the CBG tests. Although arterial blood sampling represents the gold stand for blood gas analysis, multiple studies have found that CBG analysis provides reliable and clinically useful results [15, 25].

## Conclusions

According to our results, for short surgical procedures additional PPE required during COVID-19 pandemic does not substantially affect the surgeon’s mental and physical performance. All measured data remained within the normal range at all times, and no significant differences in the attentional performance tests were noted between the special COVID-19 PPE and the regular, “standard" PPE. Our pilot study suggests that surgical procedures using COVID-19 PPE conditions are safe and do not increase the patient’s risk by adding stress to the surgical procedure.
